# Cost-effectiveness of disitamab vedotin plus toripalimab in HER2-expressing advanced urothelial carcinoma in China

**DOI:** 10.3389/fphar.2026.1809459

**Published:** 2026-07-27

**Authors:** Weicheng Lei, Zhijun Guo, Weijia He, Ya Guo, Huanjun Huang, Haoyun Chen, Zhuojia Chen, Weiting Liang

**Affiliations:** 1 Department of Pharmacy, Sun Yat-sen University Cancer Center, Guangzhou, China; 2 State Key Laboratory of Oncology in South China, Guangzhou, China; 3 Collaborative Innovation Center for Cancer Medicine, Guangdong Provincial Clinical Research Center for Cancer, Guangzhou, China; 4 Department of Pharmacy, Heyou Hospital, Foshan, China; 5 School of Pharmaceutical Sciences, Southern Medical University, Guangzhou, China; 6 Department of Clinical Pharmacy, General Hospital of Southern Theater Command, Guangzhou, China

**Keywords:** advanced or metastatic urothelial carcinoma, antibody-drug conjugates, chemotherapy, cost-effectiveness, disitamab vedotin, HER2-expresing, toripalimab

## Abstract

**Background:**

Combination therapy with disitamab vedotin (DV) and toripalimab has been proven to significantly prolong progression-free survival (PFS) and overall survival (OS) first-line in patients with human epidermal growth factor receptor 2 (HER2) expressing, locally advanced or metastatic urothelial carcinoma (UC). The economic viability of this treatment strategy remains unclear. This study systematically evaluated the cost-effectiveness of DV plus toripalimab therapy versus platinum-based chemotherapy for these patients from the perspective of the Chinese healthcare system.

**Methods:**

Based on data from the RC48-C016 clinical trial, a Markov model was established to conduct a cost-effectiveness analysis of DV plus toripalimab versus platinum-based chemotherapy in patients with advanced or metastatic UC. Cost parameters were estimated by referencing published literature or local pricing models, while utility parameters were derived from published literature. The total cost, quality-adjusted life years (QALYs) for each group, and the incremental cost-effectiveness ratio (ICER) were calculated. The ICER was compared with China’s willingness-to-pay (WTP) threshold to evaluate the economic efficiency of the two treatment strategies. Additionally, sensitivity analysis and scenario analysis were performed to verify the robustness of the established Markov model.

**Results:**

Patients in the chemotherapy group incurred a total cost of $59,329 with a corresponding gain of 1.78 QALYs. In comparison, patients in the DV plus toripalimab group had an incremental cost of $64,361 and an incremental QALY gain of 0.74, yielding an ICER of $86,954 per QALY. This value exceeded China’s WTP threshold of $40,977 per QALY. Results of sensitivity and scenario analyses indicated that the cost of DV was the most influential factor on the ICER, and the model outputs were robust. For the combination therapy to be cost-effective, a 60% price reduction for both DV and toripalimab, or a 77% price reduction for DV alone, would be required.

**Conclusion:**

DV in combination with toripalimab is not cost-effective compared with platinum-based chemotherapy first-line in the treatment of patients with HER2-expressing locally advanced or metastatic UC in China.

## 1 Introduction

Bladder cancer is a prevalent malignant tumor worldwide. In 2022, there were 613,791 (Bray et al., 2024) new global cases and 91,893 (Xia et al., 2022) new cases in China. Urothelial carcinoma (UC) is the most common subtype, accounting for approximately 90% of all bladder cancer cases,and about 11% of UC patients are initially diagnosed with locally advanced or metastatic disease (Hepp et al., 2021). Platinum-based chemotherapy was previously the standard first-line treatment for metastatic or advanced UC; however, its clinical efficacy is limited, the 5-year survival rate is only about 6%–13% (von der Maase et al., 2005; Audisio et al., 2022).

In recent years, immune checkpoint inhibitors (ICIs), including pembrolizumab, nivolumab, and atezolizumab, have been applied in the treatment of advanced or metastatic UC. However, neither ICI monotherapy nor combination therapy with chemotherapy has substantially improved outcomes in patients with advanced or metastatic UC (Galsky et al., 2020; Powles et al., 2021; van der Heijden et al., 2023).

Antibody-drug conjugates (ADCs) have seen rapid development in recent years, demonstrating tolerability and response in patients with multi-line treatments and concurrent visceral metastases and offering a new direction for systemic therapy of advanced UC. The EV-302 (Powles et al., 2024) trial demonstrated that enfortumab vedotin (EV), an ADCs targeting Nectin-4 (McGregor and Sonpavde, 2019), in combination with pembrolizumab, significantly prolonged progression-free survival (PFS) (median, 12.5 vs. 6.3 months) and overall survival (OS) (median, 31.5 vs. 16.1 months) compared with chemotherapy in patients with advanced UC. Another clinical trial, RC48-C016 (Sheng et al., 2025), showed that disitamab vedotin (DV), another ADCs targeting HER2 (Shi et al., 2022), in combination with toripalimab, also significantly prolonged PFS (median, 13.1 vs. 6.5 months) and OS (median, 31.5 vs. 16.9 months) compared with platinum-based chemotherapy in patients with previously untreated locally advanced or metastatic UC.As innovative drugs, EV and pembrolizumab failed to pass China’s national medical insurance negotiations, so their prices remain very high. Although EV combined with pembrolizumab has been proven effective in advanced or metastatic UC, it is not cost-effective in China (You et al., 2024). In contrast, both DV and toripalimab have passed China’s national medical insurance negotiations, leading to a substantial reduction in their prices. However, the cost-effectiveness of DV plus toripalimab in the treatment of previously untreated locally advanced or metastatic UC in China remains uncertain. This study aims to evaluate the cost-effectiveness of DV plus toripalimab versus chemotherapy in the treatment of patients with previously untreated human epidermal growth factor receptor 2 (HER2) expressing locally advanced or metastatic UC from the perspective of the Chinese healthcare system.

## 2 Materials and methods

### 2.1 Patients and interventions

According to the RC48-C016 (NCT05302284) study protocol, eligible patients were those with previously untreated locally advanced or metastatic UC who had centrally confirmed HER2-expressing tumors. Patients were randomly assigned in a 1:1 ratio to receiving DV 2 mg/kg plus toripalimab 3 mg/kg once every 2 weeks, or gemcitabine 1000 mg/m^2^ body surface area on Days 1 and 8 of each 3-week cycle, combined with either cisplatin 70 mg/m^2^ or carboplatin (area under the curve [AUC] = 4.5 mg/mL/min) on Day 1 of each 3-week cycle. Trial treatment was administered until disease progression, unacceptable toxic effects, or completion of the maximum number of treatment cycles (6 cycles for the chemotherapy group) occurred, whichever came first. In the chemotherapy group, 128 patients received cisplatin and 113 received carboplatin (Sheng et al., 2025).

Following disease progression, subsequent systemic anticancer treatment was initiated in 66 patients in the DV plus toripalimab group and 166 patients in the chemotherapy group. Specifically, in the DV plus toripalimab group, 45 patients received chemotherapy and 21 received ICI monotherapy. In the chemotherapy group, 75 patients received ICI combined with anti-HER2-containing therapy, 45 patients received anti-HER2-containing monotherapy, and 46 patients received ICI monotherapy (Supplementary Table S1).

In accordance with the guidelines of the Chinese Society of Clinical Oncology (CSCO) and the Supplementary Appendix of RC48-C016 study, the following subsequent treatment regimens were assumed for analysis: Chemotherapy: gemcitabine at 1000 mg/m^2^ on Days 1 and 8 of each 3-week cycle, plus either cisplatin 70 mg/m^2^ or carboplatin 4.5 mg/mL/min on Day 1 of each 3-week cycle; ICI monotherapy: tislelizumab 200 mg once every 3 weeks; ICI plus anti-HER2-containing therapy: DV 2 mg/kg plus toripalimab 3 mg/kg once every 2 weeks; Anti-HER2-containing therapy alone: DV 2.0 mg/kg once every 2 weeks. For patients (177 in DV + toripalimab group, 75 in chemotherapy group) who did not receive systemic anticancer therapy after disease progression, best supportive care (BSC) was assumed as their subsequent treatment (Chen et al., 2025).

### 2.2 Model construction

While guidelines from the National Institute for Health and Care Excellence (NICE) designate partitioned survival models (PSM) as the preferred analytical framework for health economic evaluations in oncology, we adopted a Markov cohort model in the present analysis. This modelling selection arose from two key barriers that prevented robust PSM development: first, the RC48-C016 trial did not provide independent, reliable post-progression survival data; second, crossover was observed between PFS and OS curves in the control arm, which compromised the accuracy of long-term extrapolation within a PSM framework. So, we developed a Markov model with three mutually exclusive health states (PFS, PD, Death) to evaluate the cost-effectiveness of DV plus toripalimab in patients with previously untreated locally advanced or metastatic UC. The health states transitions restricted to specific irreversible pathways (Supplementary Figure S1). GetData Graph Digitizer (Version 2.22) was employed to extract data points for PFS and OS from the Kaplan-Meier (K-M) curves presented in the RC48-C016 trial. The data extracted from the RC48-C016 study were used to reconstruct individual patient data (IPD) using the “IPDfromKM” (Liu et al., 2021) package in R (Version 4.5.1). Several parametric distributions (Exponential, Weibull, Log-Normal, Log-Logistic, Gompertz) were fitted to simulate and extrapolate the K-M curves derived from the previously reconstructed IPD, using the “survHE” (Baio, 2020) package in R. The “survHE” package generates the Akaike Information Criterion (AIC), Bayesian Information Criterion (BIC) (Supplementary Table S2), and fitting parameters for each distribution model. Lower AIC and BIC values indicate a better goodness-of-fit of the distribution model. Parameters of the best-fitting distribution (Table 1) were then applied to calculate the transition probabilities between different health states. The original and simulated survival curves were presented in (Supplementary Figures S2, S3). A 3-week cycle length and a 20-year time horizon (over 95% patients died in the time horizon) were adopted in the model, consistent with the dosing regimens and follow-up frequency reported in the RC48-C016 study.

**TABLE 1:** The best-fitting distributions and corresponding parameters used for simulating survival curves.

Curves	Distribution	Parameters
DV + T PFS	Log-logistic	Shape = 1.65, Scale = 13.79
DV + T OS	Log-logistic	Shape = 1.67, Scale = 27.21
Chemotherapy PFS	Log-logistic	Shape = 2.21, Scale = 6.42
Chemotherapy OS	Log-logistic	Shape = 1.64, Scale = 17.70

*DV*, disitamab vedotin; *OS*, overall survival; *PFS*, progression-free survival, *T* toripalimab.

### 2.3 Costs and utilities

In the cost calculations, only direct medical costs were considered, including drug costs, adverse event management costs, drug administration fees, hospitalization expenses (encompassing bed fees, diagnostic fees, treatment fees, and nursing fees), laboratory tests [hematology, blood biochemistry, Urinalysis, coagulation function, and thyroid gland function (once per treatment cycle)], HER2 test, Imaging examinations [Plain and enhanced computerized tomography (CT) of the chest, abdomen, and pelvis per 8 weeks], best supportive care (BSC), and palliative care costs. Drug prices were retrieved from the government drug procurement platform (https://gpo.gzggzy.cn/, accessed on 2026/1/11). Costs associated with adverse event management (Liu et al., 2025; Yao et al., 2025) BSC (Zhu Y. et al., 2025), and palliative care (Li et al., 2019) were derived from published literature. Test costs, drug administration fees, and hospitalization expenses were calculated based on expert opinions and local charging standards (Supplementary Table S3). For the calculation of adverse event management costs, only grade 3–5 serious adverse events (SAEs) with an incidence of ≥5% were included. According to the RC48-C016 study, the grade 3–5 SAEs with an incidence exceeding 5% were anemia, neutropenia, thrombocytopenia, γ-glutamyltransferase level increased, hypokalemia, hypoesthesia and increased lipase. The incidences of those SAEs were 5.3%, 5.8%, 0.8%, 7.4%, 5.8%, 5.3%, 5.3% in DV plus toripalimab group and 45.5%, 61.7%, 45.9%, 1.8%, 0.9%, 0%, 0.5% in the chemotherapy group. For dosage calculation, patients were assumed to have a bodyweight of 65 kg, a body surface area of 1.72 m^2^, and a creatinine clearance rate of 100 mL/min (Long et al., 2025).

According to the RC048-C016 trial protocol, DV and toripalimab were administered once every 2 weeks, while the Markov model employed a 3-week cycle length. To eliminate potential bias in total drug expenditure and fully capture discrete vial wastage arising from each independent drug reconstitution, a total-cost conservation allocation method was adopted instead of simplistic time-proportional scaling. The mean treatment duration was 34 weeks, yielding a total of N_dose_ = 34/2 = 17 actual clinical administrations. Within this timeframe, the 3-week Markov model contained N_cycle_ = ⌊34/3⌋ = 11 complete cycles. Let C_single_ represent the full cost of one biweekly dosing event, encompassing drug acquisition cost, administration fees, pre-treatment laboratory testing expenses, relevant hospitalization costs, and discrete vial wastage generated during a single reconstitution procedure. The total treatment-related expenditure for DV and toripalimab over the entire 34-week treatment period was determined as the cumulative cost of all 17 actual administrations. To preserve the real-world total drug expenditure and full cumulative vial wastage while matching the 3-week model structure, the fixed total treatment cost was evenly distributed across all complete modelled cycles. The per-cycle DV plus toripalimab treatment cost was calculated as follows: C_cycle_=(17×C_single_)/11. The cycle cost conversion ratio of 2-week treatment cycle cost to 3-week Markov cycle cost is 17/11.

Since there is no report on the quality of life of patients in the RC48-016 study, utility values for distinct health states in the model were also derived from published literature, with PFS and PD assigned utility values of 0.84 and 0.80 (Patterson et al., 2019; Vuky et al., 2020; Xie et al., 2022; You et al., 2024), respectively. Disutility associated with adverse events was incorporated into the model, with corresponding disutility values also extracted from published literature (Xiang G. et al., 2024; Xiang et al., 2024 Z.; Liu et al., 2025; Zhu K. et al., 2025). Consistent with the cost calculation methodology, only grade 3–5 serious SAEs with an incidence of ≥5% were included in the disutility analysis. The exchange rate used for converting Chinese yuan (CNY) to United States dollars (USD) was 7.01 (http://www.safe.gov.cn/, accessed on 2026/1/11).

### 2.4 Base-case analysis

TreeAge Pro Healthcare (Version 2022) was utilized for statistical analysis. Following the input of all parameters into the model, the total cost and quality-adjusted life years (QALYs) for each group were calculated, and the incremental cost-effectiveness ratio (ICER) of DV plus toripalimab combination versus chemotherapy was subsequently computed. In line with the Chinese Guidelines for Pharmacoeconomic Evaluations (Liu et al., 2022), the willingness-to-pay (WTP) threshold was set at three times China’s *per capita* Gross Domestic Product (GDP). China’s *per capita* GDP in 2024 was $13,659 (https://data.stats.gov.cn/, accessed on 2026/1/15), resulting a WTP threshold of $40,977 per QALY. The calculated ICER will be compared with thid predefined WTP threshold to assess the economic viability of DV plus toripalimab for the treatment of advanced or metastatic UC. A 5% discount rate was applied to both cost and QALY calculations in accordance with standard pharmacoeconomic evaluation practices.

### 2.5 Sensitive analysis

To confirm the robustness of the model, one-way sensitivity analysis and probabilistic sensitivity analysis (PSA) were performed. For the one-way sensitive analysis, the annual discount rate was varied within the range of 0%–8%, cost parameters were varied by ±20% relative to their baseline values, utility and disutility parameters and cycle cost conversion ratio were varied by ±10% relative to their baseline values. For PSA, 1000 Monte Carlo simulations were conducted to examine the impact on the model results when all parameters were varied in accordance with their respective probability distributions. The ranges and distributions of the parameters are summarized in Table 2.

**TABLE 2:** Parameters and corresponding sources.

Parameters	Value	Range		Distribution	Source
		min	max		
Drug costs ($)					
Disitamab Vedotin per 60 mg	542.08	433.66	650.50	gamma	Procurement platform
Toripalimab per 80 mg	115.96	92.77	139.15	gamma	Procurement platform
Tislelizumab per 100 mg	178.82	143.06	214.58	gamma	Procurement platform
Gemcitabine per 0.2 g	38.87	31.10	46.64	gamma	Procurement platform
Cis-platinum per 30 mg	2.73	2.18	3.28	gamma	Procurement platform
Carboplatin per 150 mg	22.30	17.84	26.76	gamma	Procurement platform
Cost of SAEs Management per unit ($)					
Anemia	422.22	337.78	506.66	gamma	Yao et al. (2025)
Neutrophil count decreased	358.55	286.84	430.26	gamma	Yao et al. (2025)
Platelet count decreased	933.32	746.66	1119.98	gamma	Yao et al. (2025)
γ-Glutamyltransferase level increased	1000	800	1200	gamma	Liu et al. (2025)
Hypokalemia	3,000	2,400	3,600	gamma	Liu et al. (2025)
Hypoesthesia	58.76	47.01	70.51	gamma	Xiang et al. (2024b)
Increased Lipase	221.34	177.07	265.61	gamma	Local charge
Other costs ($)					
HER-2 test per unit	271.04	216.83	325.25	gamma	Local charge
Administration per unit	8.5	6.8	10.2	gamma	Local charge
Hospitalization expenses per unit	49.11	39.29	58.93	gamma	Local charge
laboratory tests per unit	66.09	52.87	79.31	gamma	Local charge
Imaging per cycle	146.87	117.50	176.24	gamma	Local charge
Best supportive care per cycle	711	568.80	853.20	gamma	Zhu et al. (2025b)
Palliative care per unit	2085	1668	2,502	gamma	Li et al. (2019)
Utility					
PFS	0.84	0.756	0.924	beta	Patterson et al. (2019), Vuky et al. (2020), Xie et al. (2022)
PD	0.80	0.72	0.88	beta	Patterson et al. (2019), Vuky et al. (2020), Xie et al. (2022)
Disutility of SAEs					
Anemia	0.07	0.063	0.077	beta	Xiang et al. (2024a)
Neutropenia	0.2	0.18	0.22	beta	Xiang et al. (2024a)
Thrombocytopenia	0.2	0.18	0.22	beta	Xiang et al. (2024a)
γ-Glutamyltransferase level increased	0.1	0.09	0.11	beta	Liu et al. (2025)
Hypokalemia	0.04	0.03	0.05	beta	Liu et al. (2025)
Hypoesthesia	0.23	0.18	0.28	beta	Xiang et al. (2024b)
Increased lipase	0.47	0.38	0.56	beta	Zhu et al. (2025a)
Other parameters					
Discount rate	5%	0	8%	Normal	Liu et al. (2022)
cycle cost conversion ratio	17/11	1.39	1.70	gamma	Sheng et al. (2025)

*OS*, overall survival; *PFS*, progression-free survival; *SAEs*, serious adverse events.

### 2.6 Scenario analysis

A series of scenario analysis were conducted to further validate the robustness of the model.

Scenario 1: The distributions used to simulate survival curves may have a significant impact on the ICER. Therefore, the second-best fitting distributions were adopted instead of the optimal ones to simulate survival curves, aiming to examine the resultant differences in ICER.

Scenario 2: Given the substantial price gap between originator drugs and generic drugs, originator drugs were predominantly used in this study. Herein, originator drugs were replaced with generic drugs to assess the impact on ICER.

Scenario 3: DV was priced per vial (60 mg). For a patient with a body weight of 65 kg, 3 vials of DV were required per treatment cycle, resulting in 50 mg of drug waste. To identify the potential impact of this waste on outcomes, we assumed that DV is charged by milligrams and recalculated ICER to determine the resulting difference.

Scenario 4: We change the time-horizon of the study from 20 years to 15, 10 and 5 years to evaluate the impact of time-horizon on the ICER.

Scenario 5: Since the cost of DV had the most significant influence on ICER among all parameters, the prices of DV and toripalimab were adjusted to derive the critical value at which ICER equals the predefined willingness-to-pay (WTP) threshold.

## 3 Results

### 3.1 Base-case results

Base-case results showed that patients in chemotherapy group cost $59,329 and achieved 1.78 QALYs. In contrast, patients in DV plus toripalimab group spent an additional $64,361 and gained an extra 0.74 QALYs, resulting an ICER of $86,954 per QALYs, bigger than the predefined WTP threshold (Table 3).

**TABLE 3:** Base-case analysis results.

Treatment	Total cost ($)	Incr cost ($)	QALYs	Incr QALYs	ICER ($/QALYs)
Chemotherapy	59,329	​	1.78	​	​
DV + T	12,3689	63,361	2.52	0.74	86.954

*DV + T* disitamab vedotin plus toripalimab, *ICER*, incremental cost-effectiveness ratio, *Incr* incremental; *QALY*, quality-adjust life year.

### 3.2 Sensitive analysis results

**FIGURE 1:**
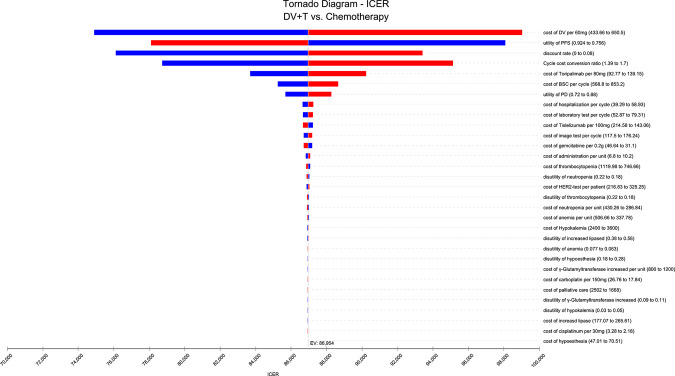
One-way sensitivity analysis (tornado diagram) for the incremental cost-effectiveness ratio (ICER). The tornado diagram summarizes the impact of varying key model inputs on the ICER of DV plus toripalimab versus platinum–based chemotherapy. Parameters were varied within prespecified ranges (cost inputs ±20%, utility inputs and cycle cost conversion ratio ±10%, and discount rate 0%–8%), while holding all other parameters constant. Bars indicate the resulting ICER range for each parameter, ordered by influence. *DV* disitamab vedotin, *PD* progressed disease, *PFS* progression-free survival.

The results of one-way sensitive analysis were showed in a tornado diagram (Figure 1). The parameter Cost of DV has the greatest impact on ICER, followed by Utility of PFS, Discount rate in sequence. With individual parameter varied within the set range, the ICER remain above $70,000 per QALYs, obviously greater than the predefined WTP threshold.

**FIGURE 2:**
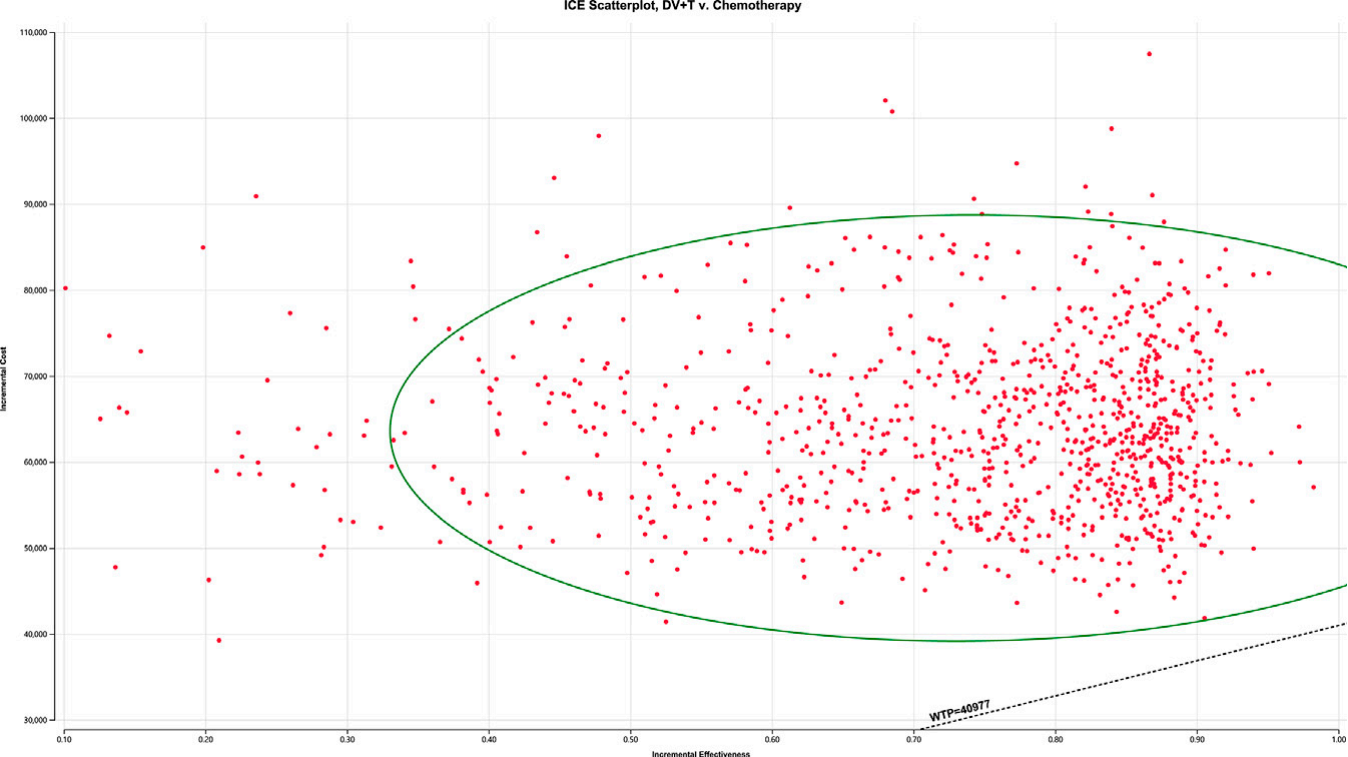
Probabilistic sensitivity analysis (PSA): cost-effectiveness plane. Scatter plot of incremental costs and incremental QALYs for DV + T versus chemotherapy generated from Monte Carlo simulation (1,000 iterations). Each point represents one simulation draw. The willingness-to-pay (WTP) threshold line indicates the maximum acceptable incremental cost per QALY; points below the line are considered cost-effective at the specified WTP. *DV + T* disitamab vedotin plus toripalimab, *QALY* quality-adjusted life-year, *WTP* willingness-to-pay.

**FIGURE 3:**
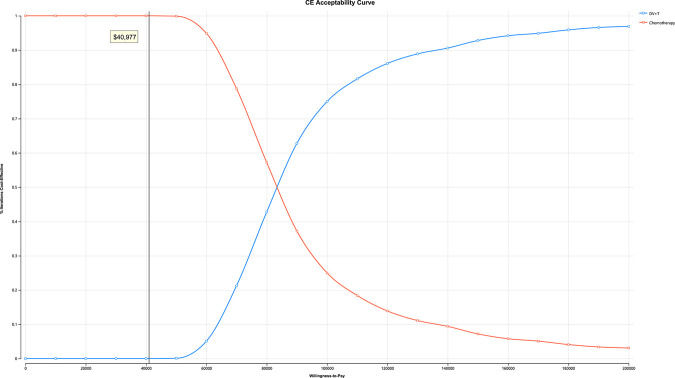
Cost-effectiveness acceptability curve (CEAC). Probability that disitamab vedotin plus toripalimab is cost-effective compared with chemotherapy across a range of WTP thresholds, derived from the PSA (1,000 iterations). The curve summarizes decision uncertainty by showing the proportion of simulations in which the net monetary benefit of disitamab vedotin plus toripalimab exceeds that of chemotherapy at each WTP value. *PSA* probabilistic sensitivity analysis, *WTP* willingness-to-pay.

The results of PSA analysis are presented in the cost-effectiveness plane Scatter plot (Figure 2) and the cost-effectiveness acceptability curve (CEAC) (Figure 3). In the scatter plot of the cost-effectiveness plane, all points fell above the WTP threshold line, indicating that DV plus toripalimab had no chance of being cost-effective. The CEAC showed that when WTP threshold was $50,000 per QALY, DV plus toriplimab had almost no probability of being economically (<0.01); when the threshold was $80,000 per QALY, the probability of DV plus toriplimab being cost-effective was approximately 50%; when the threshold increased to $170,000 per QALY, the probability of DV plus toriplimab being cost-effective exceeded 95%.

### 3.3 Scenario analysis results

Scenario analysis results are presented in Table 4. Irrespective of the adoption of different distribution models, the substitution of generic drug prices for those of originator drugs, the assumption of DV charged by milligrams or the different time-horizons, the ICER value remains substantially higher than the predefined WTP threshold ($40,977 per QALY). When all other factors are held constant, the combination therapy of DV plus Toriplimab can only achieve cost-effectiveness in the treatment of locally advanced or metastatic UC if both DV and Toriplimab are simultaneously discounted to 40% of their original prices, or if DV alone is discounted to 23% of its original price.

**TABLE 4:** Results of scenario analysis.

Scenario	Choice	ICER ($/QALY)
1, Different distributions	All survival curves follow weibull	93,493
2, Original or Generic drugs	Generic drug	88,112
3, Different charged model for DV	Charged per milligram	70,191
4, Different time-horizon	Base-case 20-year	86,954
​	15-year	89,124
​	10-year	94,475
​	5-year	115,669
5, DV and T Simultaneous price reduce	60% of base-case values	56,273
​	50% of base-case values	48,602
​	40% of base-case values	40,933
DV price reduce only	40% base-case value	50,747
​	30% base-case value	44,713
​	25% base-case value	41,696
​	23% base-case value	40,489

*DV*, disitamab vedotin, *T* toripalimab.

## 4 Discussion

For a long time, there has been a lack of effective treatment strategies for advanced urothelial UC. ADCs combined with ICIs provide a new option for the treatment of advanced UC. Both EV plus pembrolizumab and DV plus toripalimab have demonstrated a promising risk-benefit profile in patients with advanced UC. On the other hand, these innovative drugs, which are highly priced, impose a heavy financial burden on patients and the healthcare system. Therefore, cost-effectiveness analyses of these new regimens are necessary for the rational utilization of healthcare resources. Due to their high prices, EV plus pembrolizumab regimens have been proven to be uneconomical compare to chemotherapy not only in China, but also in the United States (Li et al., 2024; Meng et al., 2025; Zhu Y. et al., 2025), and the United Kingdom (Wu et al., 2025). To date, there is still no published literature on the cost-effectiveness analysis of DV combined with toripalimab for advanced UC. Owing to their inclusion in the national negotiation list of China’s medical insurance, the prices of DV and toripalimab are significantly lower than those of EV and pembrolizumab. Thus, the combination of DV with toripalimab may have chance to be cost-effective compared with chemotherapy in the treatment of advanced UC.

Based on the Markov model established using data from the RC48-C016 clinical trial in this study, the ICER of DV plus toripalimab versus chemotherapy in the treatment of advanced UC was $86,954 per QALY, which exceeds the WTP threshold in China ($40,977/QALY). Therefore, DV plus toripalimab is not cost-effective compared with chemotherapy for patients with HER2-expressing locally advanced or metastatic UC in China. Sensitivity analysis indicated that the high price of DV is the primary factor contributing to the lack of cost-effectiveness of the DV plus toripalimab regimen. Additionally, scenario analysis revealed that the per-bottle pricing model of DV further exacerbates its uneconomical nature. This might be the reason why DV plus toripalimab, as a first-line treatment for UC, has not been included in China’s medical insurance. To achieve cost-effectiveness in the treatment of locally advanced or metastatic UC, DV and toripalimab needs to be discounted to 40% of their original prices, or DV alone needs to be discounted to23% of its original price, this can provide important reference basis for medical insurance price negotiations. Data from the EV-302 and RC48-C016 trials demonstrate that the clinical benefits of DV plus toripalimab and EV plus pembrolizumab in the treatment of advanced UC are comparable; however, the total costs of these two regimens differ substantially. In this study, the total cost of the DV plus toripalimab group was $123,689, whereas the total cost for patients receiving EV plus pembrolizumab were estimated to be $375,420 (You et al., 2024) and $288,347 (Zhu Y. et al., 2025), both of which are considerably higher than those of DV plus toripalimab. Thus, although DV plus toripalimab is not cost-effective relative to chemotherapy, it may be a more cost-favorable alternative to EV plus pembrolizumab.

Only tislelizumab and toripalimab were selected as ICI agents for subsequent-line treatment in our model, whereas other guideline-recommended ICIs—avelumab, atezolizumab, nivolumab, and pembrolizumab—were excluded. The rationale is as follows: in mainland China, avelumab has not been launched to date; atezolizumab has no approved indications for UC; pembrolizumab is only approved for UC in combination with enfortumab vedotin; nivolumab is authorized solely for advanced UC alongside cisplatin plus gemcitabine. In addition, nivolumab, pembrolizumab, and atezolizumab are not covered by the national medical insurance catalogue. Considering drug accessibility and real-world clinical practice compliance, toripalimab and tislelizumab represent the only suitable options for subsequent ICI therapy.

This study possesses several notable advantages. Firstly, it represents the first cost-effectiveness analysis of DV plus toripalimab versus chemotherapy as first-line treatment for HER2-expressing locally advanced or metastatic UC in China. The findings therefore can provide valuable evidence for medical insurance policy formulation and the optimization of clinical treatment decision-making. Secondly, all patients enrolled in the RC48-C016 trial were of Asian ethnicity (100%), ensuring that the study results are highly applicable to the Chinese population.

This study also has several limitations. Firstly, due to the lack of long-term survival data from the RC48-C016 trial, we used a survival model to simulate and extrapolate the Kaplan-Meier (K-M) curves of the trial. This approach may have introduced bias into the model. To minimize such bias, the AIC and BIC were employed to select the best-fitting model. Additionally, a scenario analysis was conducted by altering the survival distribution used for simulating and extrapolating the K-M curves to confirm the robustness of the model. Secondly, when calculating the cost of adverse event management, only grade 3–5 adverse events with an incidence of ≥5% were taken into consideration. This selection criterion may lead to an underestimation of the costs associated with adverse event management. However, sensitivity analysis revealed that the costs of SAEs management had a negligible impact on the ICER. Thirdly, since the RC48-C016 trial did not report data on patients’ quality of life, the utility values utilized in this study were derived from published literature of comparable studies, which may introduce potential bias into the research results. To address this limitation, this study incorporated the reduction in utility values induced by relevant adverse reactions into the economic calculation. Furthermore, a sensitivity analysis was performed on the utility values and the utility value reduction associated with adverse reactions to verify the robustness of the study findings. Fourthly, this study adopted a 3-week Markov cycle, which created a structural mismatch with the biweekly dosing schedule of DV and toripalimab in the pivotal trial. To eliminate methodological bias arising from simplistic time-proportional cost scaling, we adopted a total-cost conservation allocation method in the revised analysis. This approach strictly preserves the total drug acquisition cost and cumulative vial wastage corresponding to the 17 actual clinical administrations delivered over the 34-week treatment duration, effectively resolving the risk of underestimated drug expenditure and artificially compressed wastage volume caused by cycle–dosing inconsistency. Nevertheless, this method entails inherent limitations. While the lifetime total treatment cost remains fully unbiased at the aggregate level, evenly distributing discrete biweekly dosing expenditures across 11 complete 3-week cycles smooths the timing of real-world resource consumption. Consequently, the model cannot fully replicate the intermittent clinical pattern of drug administration, and per-dose vial wastage events. A One-way sensitivity analysis was further performed to validate the robustness of the cost allocation strategy, wherein the 17/11 allocation ratio was varied within a ±10% range. Despite modest fluctuations in the ICER, the base-case cost-effectiveness conclusion remained unaltered, confirming the stability of our primary findings under plausible costing variations. Fifth, owing to huge regional differences in income and healthcare expenditure across China, the uniform threshold based on national *per capita* GDP is relatively imprecise. The national average conceals variations in insurance budgets and medical affordability, which may produce regionally biased results. Limited by insufficient subnational data, we applied the WHO-recommended threshold of 3 times *per capita* GDP. We acknowledge this limitation, and future studies can construct regional thresholds to improve accuracy. Finally, our model does not fully reflect China’s family-centered advanced cancer care. We only included direct medical costs and calculated QALYs without end-stage disease severity weighting. Patient quality-of-life losses, family caregiving burdens, regional policy heterogeneity and the value of clinical innovation were not quantified due to the absence of localized real-world data. Future research should develop regional thresholds and incorporate these non-medical factors to enhance model comprehensiveness.

## 5 Conclusion

Although the combination of DV and toripalimab has demonstrated promising efficacy outcomes, the regimen remains not cost-effective relative to chemotherapy for the treatment of HER2-expresing advanced or metastatic urothelial carcinoma UC in China given the current drug pricing.

## Data Availability

The raw data supporting the conclusions of this article will be made available by the authors, without undue reservation.
