# Supplementary material for: Cost-effectiveness of disitamab vedotin plus toripalimab in HER2-expressing advanced urothelial carcinoma in China
Source: Front Pharmacol. 2026 Jul 27;17:1809459. doi: 10.3389/fphar.2026.1809459 (PMC13454782; doi:10.3389/fphar.2026.1809459)
Supplement: Supplementary file 1 [file DataSheet1.docx]

**Fig. S1** Health-state transition structure of the Markov model.

Three mutually exclusive health states were included: progression-free survival (PFS), progressed disease (PD), and death. Patients entered the model in PFS and could transition irreversibly from PFS to PD and from either PFS or PD to death in each 3-week cycle over the lifetime horizon. *PD* progressed disease, *PFS* progression-free survival.

**Table S1** Subsequent treatment after first line treatment progressed

| Treatment | DV+T group (243) | Chemotherapy group (241) |
| --- | --- | --- |
| Best supportive care | 177 (72.8%) | 75(31.1%) |
| Chemotherapy | 45 (18.6%) | 0 |
| ICIs monotherapy | 21 (8.6%) | 46(19.1%) |
| ICIs plus anti-HER2-containing | 0 | 75(31.1%) |
| Anti-HER2-containing | 0 | 45(18.7%) |

*D+V* disitamab vedotin plus toripalimab, *HER2* human epidermal growth factor receptor 2, *ICIs* immune checkpoint inhibitors

**Table S2** The AIC/BIC of simulated survival curves for different distributions

| Distribution | Exponential | Weibull | Gompertz | Log-normal | Log-logistic |
| --- | --- | --- | --- | --- | --- |
| DV+T PFS curve | | | | | |
| AIC | 991.21 | 981.37 | 989.38 | 977.50 | 976.45 |
| BIC | 994.71 | 988.36 | 996.36 | 984.48 | 983.43 |
| Chemotherapy PFS curve | | | | | |
| AIC | 938.10 | 898.23 | 919.80 | 916.13 | 897.11 |
| BIC | 941.58 | 905.20 | 926.77 | 923.10 | 904.07 |
| DV+T OS curve | | | | | |
| AIC | 774.38 | 762.98 | 768.17 | 763.12 | 762.18 |
| BIC | 777.87 | 769.96 | 775.15 | 770.11 | 769.17 |
| Chemotherapy OS curve | | | | | |
| AIC | 998.31 | 988.37 | 994.24 | 997.49 | 986.93 |
| BIC | 1001.80 | 995.34 | 1001.21 | 1004.46 | 993.90 |

*DV+T* disitamab vedotin plus toripalimab, *OS* overall survival, *PFS* progression-free survival

**Fig. S2** Kaplan–Meier progression-free survival (PFS) curves and parametric model fits.

Observed PFS Kaplan–Meier curves from RC48-C016 and the corresponding fitted parametric survival functions used for extrapolation in the economic model. The best-fitting distributions were selected based on Akaike and Bayesian information criteria (AIC/BIC).

**Fig. S3** Kaplan–Meier overall survival (OS) curves and parametric model fits**.**

Observed OS Kaplan–Meier curves from RC48-016 and the corresponding fitted parametric survival functions used for extrapolation in the economic model. The best-fitting distributions were selected based on AIC/BIC.

**Table S3** Details of the local charge parameters estimation

| **Item** | **Unit price/￥** | **Amount** | **Total/￥** | **Total/$** |
| --- | --- | --- | --- | --- |
| **HER-2 test per unit** |  |  | **1900** | **271.04** |
| HER-2 test | 1900 | 1 | 1900 |  |
| **Administration per unit** |  |  | 59.61 | 8.5 |
| Centralized intravenous drug preparation | 25.88 | 1 | 25.88 |  |
| infusion apparatus | 21 | 1 | 21 |  |
| Infusion fee | 8.98 | 1 | 8.98 |  |
| Infusion solvent | 3.75 | 1 | 3.75 |  |
| **Hospitalization expenses per treatment cycle** |  |  | 344.25 | 49.11 |
| Bed fee/day | 50 | 3 | 150 |  |
| diagnosis and treatment fee/day | 28.75 | 3 | 86.25 |  |
| Nursing fee/day | 36 | 3 | 108 |  |
| **Laboratory tests per treatment cycle** |  |  | 463.28 | 66.09 |
| Blood routine test | 18.4 | 1 | 18.4 |  |
| Blood biochemistry test | 126.60 | 1 | 126.60 |  |
| urine analysis | 8.28 | 1 | 8.28 |  |
| coagulation function | 84.6 | 1 | 84.6 |  |
| thyroid function tests | 225.4 | 1 | 225.4 |  |
| **Imaging** |  |  | 2745.49 | 391.65 |
| Enhance MRI per position | 780 | 3 | 2340 |  |
| Computerized tomography | 55 | 2 | 110 |  |
| Contrast agent | 215.51 | 1 | 215.49 |  |
| Single-use connecting tube | 80 | 1 | 80 |  |
| **Management of Increased Lipase (SAE)** |  |  | 1551.6 | 221.34 |
| Blood biochemistry test | 126.60 | 2 | 253.2 |  |
| Octreotide /0.1mg | 68.11 | 12 | 817.32 |  |
| Bed fee/day | 50 | 4 | 200 |  |
| diagnosis and treatment fee/day | 28.75 | 4 | 115 |  |
| Nursing fee/day | 36 | 4 | 144 |  |
| Injection fee | 1.84 | 12 | 22.08 |  |

Imaging assessment relies on enhanced MRI, conducted every 8 weeks. The parameter “Imaging per cycle” is calculated at $146.87 per 3-week cycle.
